# Open Source 3D Printed Lung Tumor Movement Simulator for Radiotherapy Quality Assurance

**DOI:** 10.3390/ma11081317

**Published:** 2018-07-30

**Authors:** Darío R. Quiñones, David Soler-Egea, Víctor González-Pérez, Johanna Reibke, Elena Simarro-Mondejar, Ricardo Pérez-Feito, Juan A. García-Manrique, Vicente Crispín, David Moratal

**Affiliations:** 1 Centre for Biomaterials and Tissue Engineering, Universitat Politècnica de València, 46022 Valencia, Spain; dariomrxpro2@hotmail.com (D.R.Q.); dasoeg@etsii.upv.es (D.S.-E.); johannareibke@gmail.com (J.R.); elenasimarro@gmail.com (E.S.-M.); 2 Department of Radiophysics, Fundación Instituto Valenciano de Oncología, 46009 Valencia, Spain; vgonzalezper@hotmail.com (V.G.-P.); vcrispin@fivo.org (V.C.); 3 Thermodynamics Department, Universitat Politècnica de València, 46022 Valencia, Spain; riperez@upvnet.upv.es; 4 Institute of Design for Manufacturing and Automated Production, Universitat Politècnica de València, 46022 Valencia, Spain; jugarcia@mcm.upv.es

**Keywords:** respiratory movement, lung tumor, radiotherapy, arduino, cancer treatment, linear accelerator

## Abstract

In OECD (Organization for Economic Co-operation and Development) countries, cancer is one of the main causes of death, lung cancer being one of the most aggressive. There are several techniques for the treatment of lung cancer, among which radiotherapy is one of the most effective and least invasive for the patient. However, it has associated difficulties due to the moving target tumor. It is possible to reduce the side effects of radiotherapy by effectively tracking a tumor and reducing target irradiation margins. This paper presents a custom electromechanical system that follows the movement of a lung tumor. For this purpose, a hysteresis loop of human lung movement during breathing was studied to obtain its characteristic movement equation. The system is controlled by an Arduino, steppers motors and a customized 3D printed mechanism to follow the characteristic human breathing, obtaining an accurate trajectory. The developed device helps the verification of individualized radiation treatment plans and permits the improvement of radiotherapy quality assurance procedures.

## 1. Introduction

Lung cancer is the most common type of cancer in OECD (Organization for Economic Co-operation and Development) countries. It is responsible for over 1.38 million deaths annually [1]. There are currently several treatments for lung cancer, including surgery, radiotherapy, chemotherapy and palliative care. One of the most effective is radiotherapy. It is because malignant cells are eliminated via ionizing radiation and it is less invasive for the patient [2]. Nowadays, more than half of all cancer patients receive radiotherapy, either alone or in combination with surgery or chemotherapy [3]. However, radiotherapy has detrimental side effects, as healthy tissue is also affected by these usually high-dose-rate radiation beams.

Radiotherapy involves the use of controlled doses of high-intensity radiation to kill cancer cells or to reduce the size of tumors. In the case of lung cancer, radiotherapy is used as a main treatment in patients for whom surgery is impossible. It can also be applied before or after the surgery, as palliative therapy or to relieve blocked airways. Radiotherapy is generated outside the body, in a Linear Accelerator (LINAC), and is generally used to treat non-small cell tumor.

Regarding the side effects of radiotherapy, a distinction between acute and chronic effects is made. The former are those that appear during treatment and usually disappear within a few weeks, such as fatigue and skin reactions, among others. Chronic side effects appear months or even years after treatment and may be permanent.

One of the essential parts of external radiotherapy treatment is planning [4]. Before starting treatment, it is important to calculate the dose needed, the emission angles and other parameters that will allow the patient to receive the right amount of radiation without affecting adjacent areas.

Compared to other parts of the body, the lungs are in constant motion [5,6]. According to Seppenwoolde et al. the movement of the lung in this way is largely two-dimensional and it is possible to reproduce the movement of any part of the lung when the length and capacity of the breath is known.

**Figure 1:**
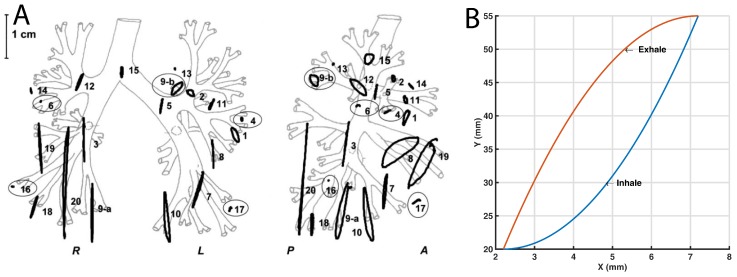
Hysteresis loop of lung tumors movement: (**A**) The orthogonal projections of the trajectories of the 21 tumors on (left) the coronal (LR-CC) and (right) the sagittal (AP-CC) plane is shown (reproduced with permission from [9]); and (**B**) One recreated hysteresis cycle that simulates the movement of the tumor is displayed.

Previous studies have shown that a human adult breathes between 16 and 20 times per minute, which means one breath (inhalation and exhalation) every 3–3.75 s [7]. Closer inspection of the lungs shows that not all points move at the same rate and, therefore, not at the same speed [8]. Nevertheless, the general movement can be equated to a hysteresis loop [9,10], as shown in Figure 1. This is why lung tumor irradiation cannot be concentrated in a fixed tumor position at each phase of the breathing cycle, and it must be applied with small margins of the target volume to cover the tumor position during the entire respiratory cycle. To irradiate the tumor, it is necessary to follow the movement of the tumor [11] to plan the radiotherapy treatment.

Good planning will reduce the amount of radiation that healthy tissues receives, while reducing the side effects. To verify the treatment of sophisticated radiation techniques in lung cancer, a parametric simulator of the tumor movement is needed.

The imaging techniques currently available, such as the 4D Computer Tomography (4DCT), acquire images synchronized with respiratory movement [12,13]. This permits to track the tumor position in each phase of the cycle and extract a custom path from patients [14,15,16,17,18,19]. These images are taken with the patient immobilized, in the same way in which the future treatment will be applied in each of the sessions.

In this work, we present a proof of concept of a lung tumor movement simulator prototype, which considers that not every human has the same breathing characteristic curve. In addition it can be adapted to any possible specific height, width and speed of breathing movement. Although there are some commercial alternatives, they are not Open-Source. The Lung Tumor Movement Simulator was entirely created using 3D printed parts in order to achieve a customizable device that can be adapted for specific cases. Furthermore, it was printed with an Open-Source 3D printer that makes it affordable for every research centre. With this device, individualized radiation treatment plans can be tested in advance.

## 2. Materials and Methods

### 2.1. Linear Accelerator

A Linear Accelerator (LINAC) is a device that is most commonly used to give external beam radiation therapy to patients with cancer. It supplies high-energy X-rays to the tumor region of the patient. These X-ray treatments can be designed to destroy cancer cells without affecting normal surrounding tissues.

The linear accelerator uses microwave technology to accelerate electrons and then allows these electrons to collide with a heavy metal target to produce high-energy X-rays [20]. These high energy X-rays are shaped as they exit the machine to target the tumor in the patient. The beam is shaped by a multi-leaf collimator that is incorporated into the head of the LINAC. The patient lies on a movable treatment couch and lasers are used to make sure that the patient is placed in the desired position. The ionizing beam is emitted from a part of the accelerator called a gantry, which can be rotated around the patient. Moreover, radiation can be delivered to the tumor from any angle, by rotating the gantry and by moving the treatment couch.

Because lungs are in constant motion, the tumor is constantly changing its position and this movement should be taken in account. This movement of the tumor is always studied before starting the treatment (Figure 1). To increase the LINACs accuracy and avoid irradiating healthy areas, there is a technique called tumor tracking [14] that is useful to analyze the movement of an internal lung tumor. Based on these tumor tracking studies, and to improve customized radiotherapy plan verification, the Lung Tumor Movement Simulator was created. It provides a good simulation of tumor movement and a good radiation measure because it holds a dosimeter in the Tip. In this case the Tip was designed to contain a Landauer OSL nanoDot™ (Landauer, Inc., Greenwood, IL, USA).

### 2.2. Electromechanical Components

To give motion to the mechanism, a pair of NEMA 17 stepper motors was used. The stepper motors have a minimum step angle of 1.8 degrees (200 steps/revolution). Each phase needs 280 mA to 7.4 V, allowing a torque of 650 g-cm (9 oz-in). To drive both stepper motors, a pair of stepper controllers DRV8825 (Texas Instruments, Dallas, TX, USA) were used, which features adjustable current limiting and six microstep resolutions (down to 1/32-step).

**Figure 2:**
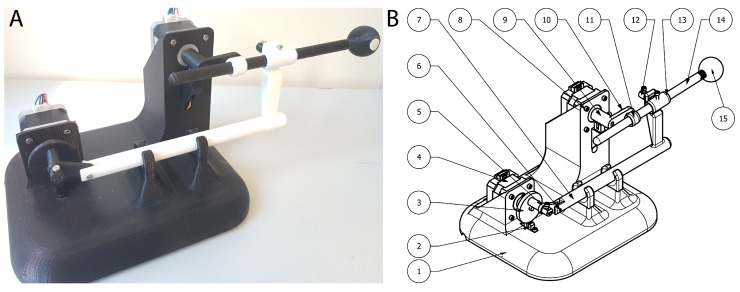
(**A**) Real Lung Tumor Movement Simulator prototype; and (**B**) Schematic parts of the prototype. See Table 1 for details.

In cyclic or rotating movements, it is important to know the starting point of the rotation. For this reason, endstops are needed. Endstops sensors are electronic components that function as switches, sending signals when an element is placed in a certain position. There are various types such as mechanical, optical and magnetic. In the prototype two optical endstops are used because they can be activated by some element of the mechanism (Figure 2, element 3 and 9). At the same time, this type of sensor avoids the possible rebounds that may appear in mechanical sensors.

### 2.3. Microcontroller

The Arduino Mega 2560 (Smart Projects, Turin, Italy & SparkFun Electronics, Boulder, CO, USA) development board is a printed circuit that allows the use of a microcontroller ATMEGA2560 (Microchip Technology Inc., Chandler, AZ, USA) [21]. Arduino is commonly used in a high variety of research fields due to its versatility and low cost [22,23,24,25].

This microcontroller controls 54 digital Input/Output pins, 15 pulse width modulation pins, and 16 analog pins, and is able to automate any system. Documentation and software are open source and available at the Arduino website. The programming software is based on the C/C++ language and the power supply of this development board can be powered through USB or main supply from 5 V to 12 V.

All of these features makes Arduino perfect for this project, but, to connect the drivers to Arduino, which control the stepper motors, an Arduino-Shield was needed. The Reprap Arduino Mega Pololu Shield (RAMPS) is a board specially designed for the use of the selected drivers. This can control up to five stepper motors and support the connection of various endstops. The RAMPS board can only be used together with an Arduino Mega 2560 board on which it is placed by inserting the male connectors of the RAMPS into female Arduino pins.

### 2.4. Manufacture and Material

The entire project was thought to be printable in any commercial or Open-Source 3D printer. A Prusa i3 MK2 with a precision of 50 microns per layer height, and a printing surface of 10,500 cm${}^{3}$ (25 × 21 × 20 cm or 9.84 × 8.3 × 8 in) was used to print all of the parts. The entire prototype was manufactured with poly(lactic acid) (PLA) material. This material was chosen because it achieves the specifications that are need for the prototype [26]. It is light and strong enough to enable the mechanism to function smoothly. Furthermore, it is biodegradable and environmentally friendly.

## 3. Results

### 3.1. Prototype Design

**Figure 3:**
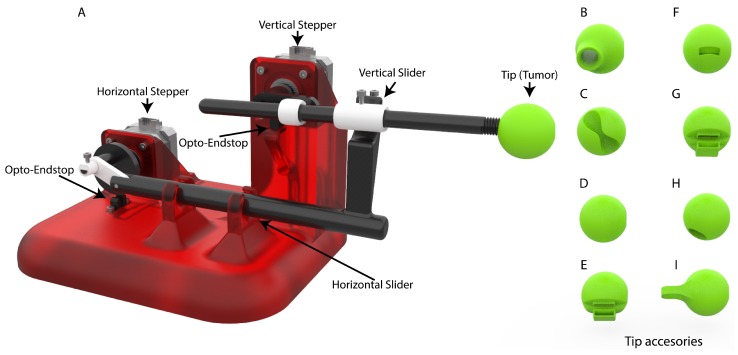
3D model of the Lung Tumor Movement Simulator. Main design of the prototype *A* and eight tips model that were designed to give versatility to the model. Tips *D*–*H* were designed with the purpose of holding a nanoDot™radiation dosimeter. Tips *B*, *C* and *I* were designed to hold a test tube with contrast fluids for other purposes.

During the design of the Lung Tumor Movement Simulator (LTMS) (Figure 3), several mechanical designs were studied and proposed; in the end, a design composed of two sliders was chosen.

This design is formed by two stepper motors, which move the mechanical elements (Figure 2) to achieve the desired movement. There is an upper motor, which controls the upwards and downwards motion of the Tip that simulates the tumor position. Then, there is a lower motor which controls the of pushing forward and backward the Tip, which represents the targeted tumor. The entire prototype is composed of 15 different parts. Their dimensions and weights are shown in Table 1.

The biggest challenge during the design was how to achieve the synchronization of the two stepper motors in order to obtain an accurate path. This problem was overcome with the addition of two optical endstops. These endstops were placed underneath each stepper motor and a protuberance on each axis is what triggers the optic mechanism.

### 3.2. Movement and Path Simulation

**Figure 4:**
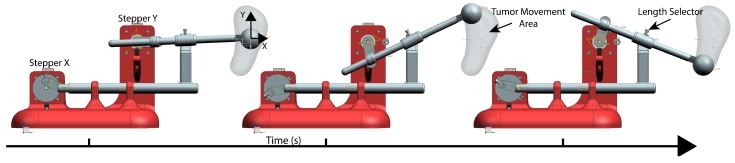
Virtual simulation of the desired area to cover with the LTMS in order to validate the viability of the system design.

To validate the prototype mechanism, it was necessary to evaluate the mobility of the whole system as shown in Figure 4. By using the feature of Movement Simulation from Unigraphics NX 11 (Siemens, Berlin, Germany), the Tip trajectory was simulated taking into account gravity, friction and speed limits.

During the virtual simulation, it was observed that the Tumor Movement Area, which is shown in Figure 4, was the expected area for the tumor movement cycle. Furthermore, the simulation was done with the same speed for the two stepper motors and one specific mechanical configuration; however, if the speed or the length of the mechanical transmission were modified, the height and width of the desired area would be modified.

### 3.3. Synchronizing the Movements

**Figure 5:**
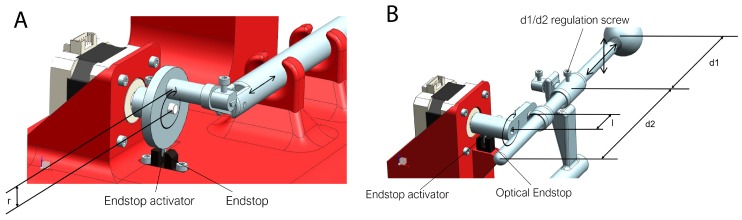
(**A**) Horizontal stepper motor; and (**B**) vertical stepper motor. See main text for details.

The rotation speed of the primary motor (Figure 5A), which is the operator of the horizontal movement, was fixed at a speed that depends on the respiratory frequency of the patient, so that a complete turn is made with the same duration as breathing is performed.

To reconstruct the hysteresis cycle, the second stepper motor (Figure 5B), which controls vertical displacement, continuously modifies the rotation speed throughout the cycle. This cycle is divided into six parts, which corresponds to a 60-degree turn of the motor. The average speed in the set of the six sections is equal to the other engine to achieve a synchronized movement and a complete turn in the same time. Therefore, the different speeds on this engine are time dependent.

The parametric variables that affects the Tip (simulated tumor) trajectory are shown in Figure 5. The simulated tumor trajectory can be adjusted in several ways:

- In width: The width can be modified by changing the distance “*r*”.
- In height: To change the height of the movement (amplitude), it is necessary only to release a little screw that is at the joint of the vertical slider (Figure 5B) and to move the rod forward or backward. This is represented by distances “*d*1” and “*d*2” (Equation (1)).
- In timing: In the serial console, the breathing time can be set to move at the same frequency as the patient does.
  (1) $$\begin{array}{cc} h & =2\cdot \left(\frac{d1\cdot l}{d2}\right) \end{array}$$

As a result of changing the distances, the output path is defined by Equations (2) and (3). These equations describes the Tip trajectory during the inhalation and exhalation.

(2) $$\begin{array}{cc} Y_{inhale} & ={\left(\left(\sqrt{\frac{h}{{\left(2\cdot r\right)}^{2}}}\right)\cdot x\right)}^{2} \end{array}$$

(3) $$\begin{array}{cc} Y_{exhale} & =\left(-{\left(x-\left(2\cdot r\right)\right)}^{2}+{\left(2\cdot r\right)}^{2}\right)\cdot \sqrt{\frac{h}{{\left(2\cdot r\right)}^{2}}} \end{array}$$

### 3.4. Path Verification

**Figure 6:**
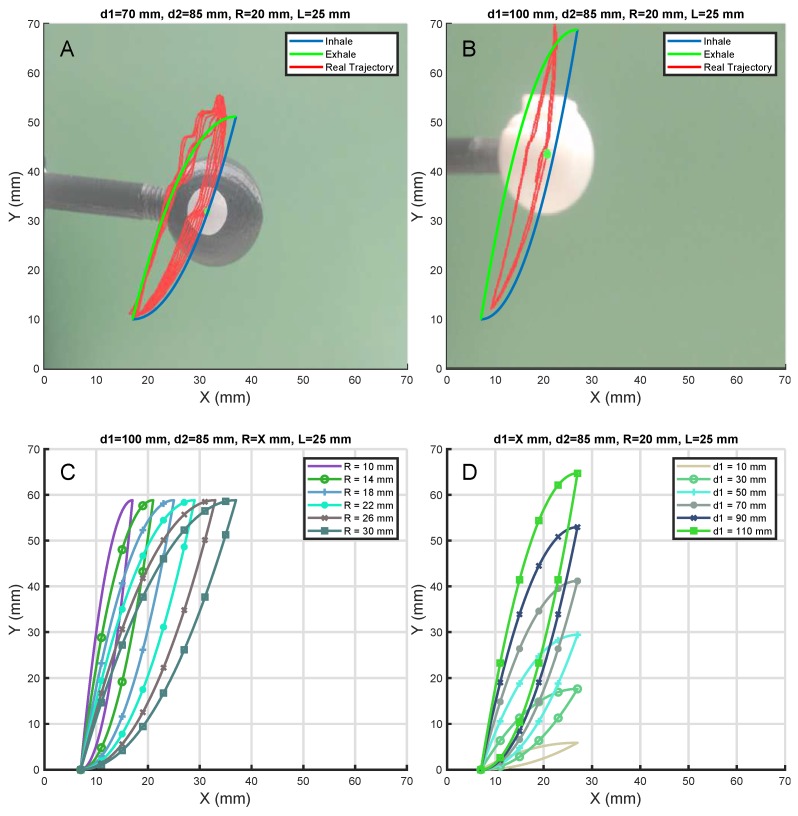
(**A**) Setup for a wider tumor trajectory is shown. (**B**) Setup for higher tumor trajectory is shown. (**C**) All possible trajectories by modifying the *r* parameter are displayed. (**D**) Possible trajectories by modifying *d1* parameter are displayed.

To check the precision of the LTMS prototype, the movement was tracked (Figure 6A,B) by creating a tracking workflow with Bonsai software [27]. Bonsai is a visual programming language that allows a modular, high-performance, open-source visual programming framework for the acquisition and online processing of data streams. It permits real time data acquisition and processing among several interfaces.

A real-time data analysis was performed to constantly track the position in X and Y of the Tip, while the prototype is running the breathing cycle. In this Bonsai workflow, the camera input with the overlapped tracking were recorded, as well as the X and Y positions of the Tip plus a time-stamp.

This tracking is performed by transforming the acquired image from the camera to a Hue, Saturation, Value (HSV) image. A white sticker was attached to the Tip (sphere), which is shown in Figure 6A,B, and by means of establishing a HSV threshold it was possible to discriminate the sticker contours against the Tip (simulated tumor). Then, the largest binary region was found, which corresponded to the sticker, and the centroid of it was detected. These centroid coordinates correspond to the X and Y position of the simulated tumor.

The resulting curves of the prototype and program differ slightly from the desired hysteresis loop; even though the lower part matches well, the upper area deviates a maximum of 5 mm from the desired position, as shown in Figure 6A,B. Several tests were made with the system during 20 cycles. It deviates in the two first cycles until the synchronization process is done. After these two cycles the deviations were smaller than 1 mm in the higher setups and 4 mm in the wider setups. However, in future versions the above mentioned errors will be minimized by improving the mechanical design.

There are several combinations that could be set up with the prototype, and depending on which of the transmission components is used it is possible to achieve all of the combinations that are shown in Figure 6C,D.

**Figure 7:**
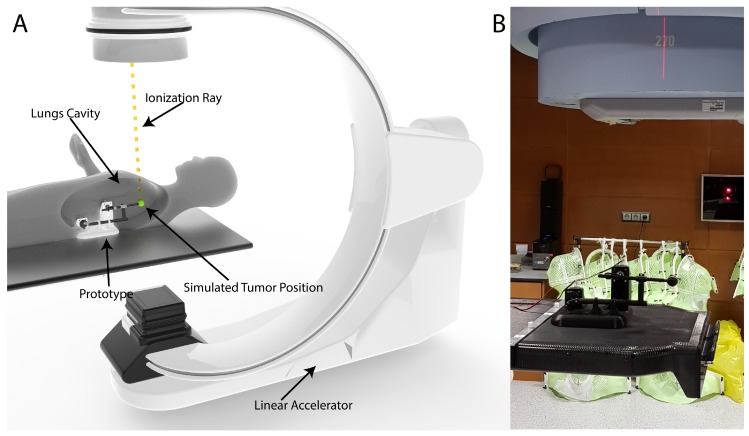
(**A**) Virtual reconstruction of the location where the prototype should be placed to simulate the tumor path is shown; and (**B**) the real Lung Tumor Movement Simulator placed inside a LINAC.

The 3D Printed Lung Tumor Movement Simulator for radiotherapy quality assurance has been presented (Figure 7) to a group of expert radiation oncologists and medical physicists from the Instituto Valenciano de Oncología, Valencia, Spain.

## 4. Discussion

In the radiotherapy field, movement of tumors due to the respiratory cycle makes treatment difficult and, for this reason, an area of research has focused on treatment planning [5,6,11]. There are devices that can be used to guarantee the quality and quantity of received radiation doses, which are commonly named phantoms. In this way, in the most complex cases, doctors and radiologists will perform a priori tests that ensure maximum precision before the treatment.

Although there are existing commercial devices such as QUASAR (Modus Medical Devices Inc., London, ON, Canada), Respiratory Gating Platform (Standard Imaging Inc., Middleton, WI, USA) and Dynamic Thorax Phantom (CIRS Inc., Norfolk, VA, USA), these alternatives are closed source and difficult to customize. The creation of this prototype based on 3D printing and Open-Source is intended to serve as a basis for the expansion of these devices for research purposes.

The Respiratory Motion Phantom from QUASAR is a commercial device developed by Modus QA, which, in its latest version, reproduces in two dimensions the respiratory movement of patients for use in radiotherapy [28]. It is useful for testing treatments, the correction of these tests and the commissioning of the implementation of new systems. However, this device is a licensed product that cannot be customized, and it is not accessible for researchers. The aim of this project is to pave the way for researchers to improve the treatment simulations in a cost-effective way. In addition, it facilitates the creation of parametric components, which allow the simulated tumor path to be changed. One of the strongest skills of this prototype is that all of the mechanisms are 3D printed and they can be modified as the researcher desires.

## 5. Conclusions

This project began with the idea of creating an Open-Source prototype that would follow the hysteresis loop of the movement of a human lung during a respiration cycle to track the movement of a tumor. We present an Open-Source Lung Tumor movement simulator, which is 3D printed for each patient and for each treatment. This prototype is completely customizable, and it paves the way for researchers, radiologists and nuclear medicine physicians to improve the radiation therapy for quality assurance procedures and outcomes.

This Lung Tumor Movement Simulator is programmable with the amplitude and frequency specific to each treatment and patient. These data are obtained thanks to the images obtained by techniques such as the 4D-CT and are translated through the serial interface that connects the computer and the hardware (drivers, motors, etc.). In the prototype, there is the possibility of introducing a dosimetric film or an ionization chamber to measure the dose of radiation absorbed. Furthermore, the Lung Tumor Movement simulator is cost-effective, because it is almost entirely 3D printed and the electronic components, as well as the motors, are not expensive.

The results of building and testing the new lung movement prototype are very promising. It has been shown that it is possible to create a simple and cost-effective machine to simulate the movement of a tumor in the lungs based on additive manufacturing. The prototype is ready to be tested and there are plans to undertake customized radiotherapy verification and research in a radiotherapy machine. In addition, all the schematics, parts and firmware are available at dmoratal.webs.upv.es/research.

## Figures and Tables

**Table 1:** Prototype parts.

PART (Figure 2)	Description	Quantity	X (mm)	Y (mm)	Z (mm)	Weight (g)	Material
1	Base	1	209.50	180.00	145.00	550.00	PLA
2	Endstop	2	-	-	-	-	-
3	Horizontal wheel	1	45.10	40.00	51.00	9.00	PLA
4	Stepper NEMA 17	2	42.00	42.00	38.00	285.00	PLA
5	Union bar	1	15.70	36.60	4.50	3.00	PLA
6	Cylinder pin	1	2.00	3.00	30.00	2.00	Stainless Steel
7	Horizontal bar	1	200.00	26.00	85.50	28.00	PLA
8	Screw M3 x 6	15	-	-	-	5.00	Stainless Steel
9	Vertical bar	1	22.00	44.90	30.00	6.00	PLA
10	Short retainer	1	4.00	10.90	9.10	1.00	PLA
11	Stem guide	1	33.00	13.40	16.20	3.00	PLA
12	Long retainer	2	9.00	10.90	9.10	2.00	PLA
13	Stem support	1	22.00	44.90	30.00	6.00	PLA
14	Stem	1	200.10	10.00	14.90	12.00	PLA
15	Tumor sphere (Tip)	1	29.20	30.00	30.00	8.00	PLA
